# Glial Activation in the Thalamus Contributes to Vestibulomotor Deficits Following Blast-Induced Neurotrauma

**DOI:** 10.3389/fneur.2020.00618

**Published:** 2020-07-15

**Authors:** Michelle R. Dickerson, Zachary Stephen Bailey, Susan F. Murphy, Michael J. Urban, Pamela J. VandeVord

**Affiliations:** ^1^Department of Biomedical Engineering and Mechanics, College of Engineering, Virginia Tech, Blacksburg, VA, United States; ^2^Salem VA Medical Center, Salem, VA, United States

**Keywords:** thalamus, amygdala, blast, vestibulomotor, microglia, astrocytes, traumatic brain injury

## Abstract

Vestibular impairment has become a frequent consequence following blast-related traumatic brain injury (bTBI) in military personnel and Veterans. Behavioral outcomes such as depression, fear and anxiety are also common comorbidities of bTBI. To accelerate pre-clinical research and therapy developments, there is a need to study the link between behavioral patterns and neuropathology. The transmission of neurosensory information often involves a pathway from the cerebral cortex to the thalamus, and the thalamus serves crucial integrative functions within vestibular processing. Pathways from the thalamus also connect with the amygdala, suggesting thalamic and amygdalar contributions to anxiolytic behavior. Here we used behavioral assays and immunohistochemistry to determine the sub-acute and early chronic effects of repeated blast exposure on the thalamic and amygdala nuclei. Behavioral results indicated vestibulomotor deficits at 1 and 3 weeks following repeated blast events. Anxiety-like behavior assessments depicted trending increases in the blast group. Astrogliosis and microglia activation were observed upon post-mortem pathological examination in the thalamic region, along with a limited glia response in the amygdala at 4 weeks. These findings are consistent with a diffuse glia response associated with bTBI and support the premise that dysfunction within the thalamic nuclei following repeated blast exposures contribute to vestibulomotor impairment.

## Introduction

Neurosensory deficits such as vestibular impairment are a frequent outcome following traumatic brain injury (TBI) and if not treated can lead to long-term disability (1). In military populations, more than 25% of the Veterans are suffering from closed head injuries due to blast exposures during combat (2, 3). Transference of blast wave energy into the brain results in neurological deficits leading to the diagnosis of blast-related traumatic brain injury (bTBI). There is a growing concern that there may be detrimental effects within the brain following multiple low- to medium-level blast exposures during military training and combat. The neurosensory sequelae following blast exposure can include auditory, sleep, vestibular and visual impairments (4–7). These neurosensory conditions have become increasingly prevalent in military personnel exposed to blast events and are a common comorbidity of bTBI. Acute vestibulomotor deficits have been noted in 98% of patients diagnosed with bTBI and 72% of these patients report long-term vestibular impairment (8, 9). Clinical manifestations of vestibular damage includes motor impairments leading to imbalance, motion intolerance, postural instability and dizziness (1, 10). In most cases, there are also significant behavioral concerns that overlap with blast-related impairments such as anxiety, attention, fear, memory, and problem-solving deficits (11–13). Due to their unique combat experiences, Veterans face many long-term health challenges that result from the blast trauma including neurosensory deficits.

The transmission of neurosensory information often involves a pathway from the cerebral cortex to the thalamus (14, 15). This indicates that the thalamus serves crucial integrative functions within vestibular processing. Peripheral vestibular stimulation has been shown to cause strong activation within the thalamus, with the ventrolateral nuclei (VL), laterodorsal nuclei (LD), and central medial nuclei (CM) of the thalamus receiving inputs from the bilateral superior vestibular nucleus (SuVN) and the contralateral medial vestibular nucleus (MVN). The VL, LD, and CM project to the primary motor and premotor cortices suggesting that the circuitry between these nuclei represents a major vestibulomotor pathway. A study in rats showed that lesions in the LD impair spatial learning and memory, suggesting that the LD is part of novel processing involved in spatial orientation and learning to sensory cues (16). Since the CM receives inputs from various vestibular nuclei, lesions in the CM have been linked to impairments in working memory and motor control (17).

The influence of vestibular stimulation on behavior can be mediated through the projections of nuclei from the vestibular system, through the thalamus, into amygdala cells (18). The amygdala is known to integrate and process information pertinent to reward and emotions such as fear and anxiety (19). Specifically, the basolateral amygdaloid complex (BLA) integrates information regarding fear and anxiety-inducing stimuli, regulating emotional and behavioral responses (20). Furthermore, the BLA receives sensory information, such as vestibular outputs, through axons networking through the superior and lateral vestibular nuclei, which then project through the thalamic nuclei to the BLA (21). Evidence linking pathological dysfunction of the amygdala and thalamus to sensory impairments is important to further understanding the mechanisms associated with bTBI.

Studies have demonstrated lesions within the vestibular nuclei, the thalamic nuclei, and the BLA correlate to vestibulomotor and stress induced deficits (22–24). However, an understanding of how the glial cells (astrocytes and microglia) contribute to the morbidities in these specific brain regions is lacking. Astrocytes are the most numerous cells in the human central nervous system (CNS) and carry out many homeostatic functions crucial for normal brain function. Astrocytes associated with injured tissue often termed reactive or gliotic astrocytes, are characterized by profound changes in protein and gene expression leading to hypertrophy, increased expression of intermediate filaments [glial-fibrillary acidic protein (GFAP), nestin and vimentin] and increased proliferation (25, 26). These changes ultimately result in homeostatic deficits, including dysregulation of critical ions and neurotransmitter uptake capacities, contributing to neuropathology. Reactive astrocytes are also characterized by a combination of structural and functional changes, which include thickening and retraction of primary, secondary and tertiary processes (27, 28). It is these fine processes in a healthy brain that are intimately associated with over 90% of functional synapses in the CNS (29). Accumulating evidence from preclinical and clinical studies suggest reactive astrocytes contribute to the TBI sequelae (30, 31). Microglia compose of approximately 10% of the total glia of the brain and function as the innate immune system in the CNS (32). They are the first line of defense, playing a critical role in neuroinflammation following injury. Microglia become activated adapting both pro- and anti-inflammatory phenotypes which produce high levels of cytokines and oxidative metabolites that are important in phagocytic activity that eliminate extracellular debris, apoptotic cells, and increases tissue remodeling (33). Additionally, it has been hypothesized that a larger number of pro and anti-inflammatory microglia would be located around traumatic lesions, and take on morphological changes in order to respond to these lesions (34, 35). Microglia are known to convert from a “healthy” ramified shape to a reactive hypertrophic, “bushy” morphology, or become “rod-like,” with thin somas and polarized processes aligning adjacent to neuronal processes (36, 37). Activated microglia have also been associated with an amoeboid morphology that further aids in phagocytic properties, which either lead to cumulative neuronal loss, or promote neuroplasticity, and axonal regeneration (38).

There is significant clinical and preclinical support for the premise that blast exposure leads to neuroinflammation. Gill et al. reported finding elevated serum levels of IL-6 and TNF-alpha acutely in a population of military personnel exposed to a blast insult (39). A report by Rusiecki et al. measured serum levels of pro- and anti-inflammatory cytokines pre- and post-deployment of those who had been diagnosed with mild and moderate bTBI (40). They found chronic changes in several inflammatory markers (MMP3, IL-1α, IL-4, IL-6, and IL-8) indicating a long-term response to blast exposure. Preclinical studies not only show elevated levels of cytokines but extend to histological measures of neuroinflammation and reactive gliosis within animals exposed to blast events (41–46). Collectively, these studies depict a significant contribution of neuroinflammation to the enduring complications of bTBI. Identifying the mechanisms that contribute to the pathological changes in the brain that link to these symptoms remains complicated. Limited attention has been given to vestibular injuries associated with bTBI. Blast waves are known to cause inner ear damage but recent debates question whether the injury is more widespread, and whether various brain regions are being affected by bTBI contributing to neurosensation deficits (47, 48). Arun et al. found significant neuromotor impairments occurring up to 6 months following repeated blast exposures in rats (49). As neurobehavioral deficits are being presented following blast exposure, identifying and studying the pathological changes that contribute to these shortfalls is imperative. We aimed to characterize neuropathological changes within the thalamus and amygdala following repeated blast exposures, thus providing more data to assist the mechanistic understanding of the vestibular impairment that presents clinically following a blast injury.

## Materials/Methods

### Animals and Blast Exposure

The study described herein was carried out in accordance with experimental protocols approved by the University Institutional Animal Care and Use Committee at Virginia Tech. Prior to any experimentation, male Sprague Dawley rats (Envigo, Dublin, VA, USA) weighing approximately 250–300 g were acclimated for several days (12 h light/dark cycle) with food and water provided *ad libitum*.

**Figure 1:**
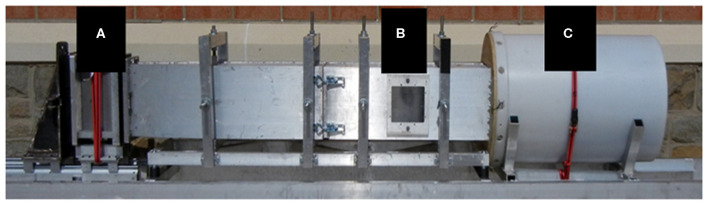
The ABS was used to re-create free field blast exposures. Acetate membranes are passively ruptured following pressurization using helium gas in the driver section **(A)**. The blast wave reaches the animal located in the test section **(B)**, and is dissipated in the end-wave eliminator **(C)**.

The blast wave was generated using a custom Advanced Blast Simulator (ABS) (200 cm × 30.48 cm × 30.48 cm) located at the Center for Injury Biomechanics at Virginia Tech University. The ABS consisted of three distinct sections to create, develop, and dissipate the blast wave (Figure 1). The blast wave developed following a helium-driven rupture of calibrated acetate membranes. The passive end-wave eliminator was located downstream of the test location to facilitate the dissipation of the blast wave through a series of baffles. As a result, the test location was exposed to a single peak overpressure representing a free-field blast exposure. Pressure measurements were collected at 250 kHz using a Dash 8HF data acquisition system (Astro-Med, Inc., West Warwick, RI, USA). Analysis of pressure profiles was conducted using a custom MATLAB script to calculate impulse and duration of the positive and negative phases and rise time. Peak overpressure was determined using the Rankine—Hugoniot relations and observed wave speed at the animal test location within the ABS.

Prior to blast exposure, animals were anesthetized with 3% isoflurane and placed in the ABS. Each animal was supported in the prone position inside the ABS facing the oncoming shock front using a mesh sling. The sling was designed to minimize flow hindrance and isolate primary blast injury by eliminating acceleration/deceleration injuries. Animals were exposed to three blasts (16.62 psi ± 2.27 psi) separated by 1 h each (3 × 1 h) (*n* = 10). There was also a sham group (*n* = 10) that received all the same procedures with the exception of blast exposure. Following the sham or blast procedures, animals were observed through the recovery stages of injury and anesthesia.

## Behavioral Assessments

### Accelerating Rotor Rod Task (RR)

Sensorimotor coordination and motor learning post-blasts were assessed using the RR (San Diego Instruments, San Diego, CA). Pre-training sessions were completed before blast exposure to ensure that animals were able to adequately perform the task and that all motor deficits would solely be due to bTBI and subsequent injury progression. In the pre-training sessions, the animals were taught to stand on the stationary rod. Once this was achieved, the rod was turned on so the animals would learn to walk at constant speeds between 3 and 21 revolutions per minute (RPM). The animals were placed back on the rod upon falling. The animals were also introduced to the accelerating protocol and a baseline reading was obtained. During testing, the animals were placed on the RR which accelerated 3 RPM every 12 s, starting at 3 RPM and finishing at 30 RPM. The maximum amount of time allotted for each trial was 120 s. Latency to fall, total distance traveled, and maximum RPM was recorded for each trial using the manufacturer's software. The RR task was performed 1 and 3 weeks following blast exposure, with the animal performing the task for a total of three trials at each time point.

### Open Field Test (OFT)

Blast induced anxiety-like behavior was measured using the OFT. The animal was placed in an arena (80 cm^2^) in a low-light room. The animal was allowed to explore the arena for 5 min. The investigator was not present inside the room at any point throughout the trial. Three-point tracking was performed using EthoVision XT and included tracking of the tip of the nose, center of the body, and base of the tail (Noldus Information Technology, Leesburg, VA, USA). Each trial was recorded at 30 frames per second and proper tracking was confirmed by an investigator blind to treatments. Anxiety is measured as thigmotaxic behavior within the OF environment (50). Therefore, the fraction of time spent along the walls of the arena was calculated and used to represent anxiolytic behavior. Locomotor function was also measured in the open field arena by measuring the total distance traveled. The OF test was administered prior to blast exposure then biweekly following blast exposure for the 1 month study.

## Immunohistochemistry (IHC)

Four weeks following blast exposure, animals were euthanized by transcardial perfusion of saline and 4% paraformaldehyde. Following perfusion, brains were collected and stored in 4% paraformaldehyde fixative solution. After 24 h in the fixative, whole brains were cryoprotected in a 30% sucrose solution for tissue sectioning preparation. Once whole brains were completely submerged in the sucrose solution (~48 h), tissues were then embedded in Tissue-Tek optimal cutting temperature (OCT) embedding medium (Sakura Finetek USA, Inc., Torrance CA) and frozen at −80° for cryostat processing. Brains were sectioned at 40 μm in the coronal plane and sections including the CM, LD, VL, and BLA were chosen (~-3.00 mm posterior from Bregma), with two random sections placed per slide for staining. Samples were rinsed three times with phosphate-buffered saline (PBS) and were permeabilized in PBS with 0.3% Triton (PBX) for 30 min at room temperature. The samples were then incubated in 2% bovine serum albumin (BSA) in PBS for 1 h at room temperature. Sections were incubated for 16–18 h at 4°C with primary antibodies glial fibrillary acidic protein (GFAP. 1:500; Invitrogen, Carlsbad, California), and ionized calcium-binding adaptor molecule 1 (IBA-1, 1:300; Biocare Medical, Concord, California). The following day, sections were washed three times for 5 min in PBX and incubated for 1.5 h at room temperature with secondary antibodies (Alexa Fluor 555 anti-rabbit IgG antibody and Alexa Fluor 488 anti-mouse IgG antibody; Invitrogen, Carlsbad, California). After three more 5 min PBX washes, samples were mounted and cover slipped with Slow Fade Reagent with DAPI (Invitrogen, Carlsbad, CA). Sections were then imaged under a Zeiss fluorescence microscope at 20X magnification.

To provide a comprehensive analysis of the glial pathology, we quantified four specific parameters using ImageJ software; area fraction, count per area, integrated density of fluorescence and mean area per cell. Area fraction quantifies the percentage of positive signal within the region of interest. Count per area represents the total number of positive cells divided by the area. Integrated density of fluorescence measures the level of fluorescence intensity in the positive signal using gray pixel intensity. Mean area per cell provides detail to the average cell soma size normalized to the area, giving the average area of the cell. Count per area and mean area per cell were completed by using the “analyze particles” function with a pixel area size threshold of 0.004 to exclude small pixel noise and extract objects of interest. Mean brain region values were derived from a minimum of four images for each animal per stain.

## Statistical Analysis

All statistical analyses were performed using GraphPad Prism version 8 (GraphPad Software, La Jolla, CA). Statistical differences between groups were assessed by the student's *t*-test. Statistical differences between groups and multiple time points were assessed by two-way ANOVA with repeated measures applying *post-hoc* tests where appropriate. Further analysis of significance and variability was done by calculating the effect size between treatment groups (ω^2^). The Shapiro-Wilk test and Levene's test were used to verify assumptions of normality and equality of variances, respectively. Data that did not pass normality or equal variance assumptions were assessed using either Welch's correction *t*-test or Mann-Whitney's non-parametric test. Data were considered statistically significant with *p* < 0.05 and trending at *p* < 0.1. All histology data was normalized to respective shams. All data is represented as the mean ± standard error of the mean, or SEM.

## Results

### Blast Event and Animal Recovery

Blast Animals (*n* = 10) were exposed to three blast events 1 h apart. Blast wave parameters are described in Table 1. Sham animals (*n* = 10) were exposed to all procedures with the exception of the blast exposures. Following exposures, no obvious external signs of injury were discernible. Over the 4 week period, there was no significant difference in the weights observed in the blast group when compared to the sham group. The average weight of the repeated bTBI animals was 361.8 g ± 10.0, while the sham group average was 349.9 g ± 15.7. The percentage of weight gain for the blast group was 1.24% when normalized to shams.

**Table 1:** Summary of blast wave characteristics.

**Treatment**	**Peak pressure (psi)**	**Duration (ms)**	Impulse (psi*ms)	**Rise time (ms)**
3 × 1 h blast	16.62 ± 2.27	2.25 ± 0.10	12.06 ± 2.64	0.049 ± 0.036

*Sprague Dawley rats (male, 250–300 g) were anesthetized and exposed to three blast insults separated by 1 h each. Sham animals underwent all procedures with the exception of the blast insult. The average peak pressure resulted in a blast wave magnitude of ~17 psi which induces a mild TBI in rodents. Results are represented as Mean ± SEM*.

### Blast-Induced Vestibulomotor Deficits

**Figure 2:**
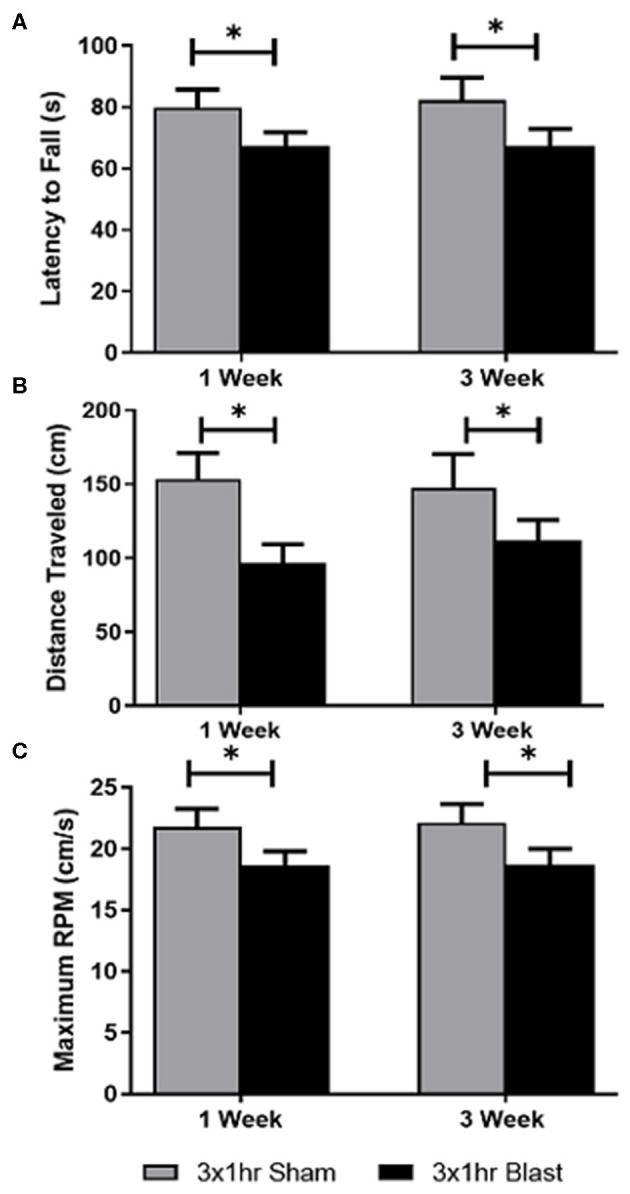
Vestibulomotor function was impaired following blast exposure. Using the accelerating rotor rod task; latency to fall, total distance traveled, and maximum RPM were quantified at 1 and 3 weeks following repeated blast exposure. A two-way ANOVA with repeated measures indicated significant differences in the three parameters. **(A)** A significant decrease in latency to fall in blast animals compared to shams was observed. No significance was found in the interaction between time and blast, or in time itself. The blast animals spent less time on the beam before falling, suggesting vestibulomotor impairment following blast injury. **(B)** A significant decrease in the distance traveled in blast animals compared to shams was also observed. No significance in the interaction between time and treatment, or in time itself was found. These decreases suggests motor impairment due to postural instability, dizziness, or balance issues. **(C)** Blast animals operated on the accelerating rotor rod at a slower RPM than sham animals. These findings were found to be significant. No significance in the interaction between time and blast or in time itself was found. *p < 0.05. Mean ± SEM.

The accelerating RR task is an established test that is an effective measure of motor function and balance impairments in rodents (51–53). Animals exhibiting motor deficits show a decrease in time on the RR beam (latency to fall), decreased distance traveled, and decrease maximum RPM on the beam compared to their sham counterparts. Results of the repeated blast exposures on RR performance are shown in Figure 2. From the repeated measures ANOVA, the blast injury had a significant effect on RR performance at both 1 and 3 weeks. Repeated bTBI animals showed a significant decrease in latency to fall (*p* < 0.05) compared to the shams, with an effect size of 0.11 (Figure 2A). Additionally, the repeated bTBI group showed a significant decrease in distance traveled and maximum RPM at both time points (Figures 2B,C), with the effect size for both parameters also found to be 0.11. There was no interaction between time and blast for latency to fall, distance traveled, and maximum RPM on the RR. Time itself also did not have a significant effect on either parameter measured for RR.

### Anxiety-Like Behaviors

**Figure 3:**
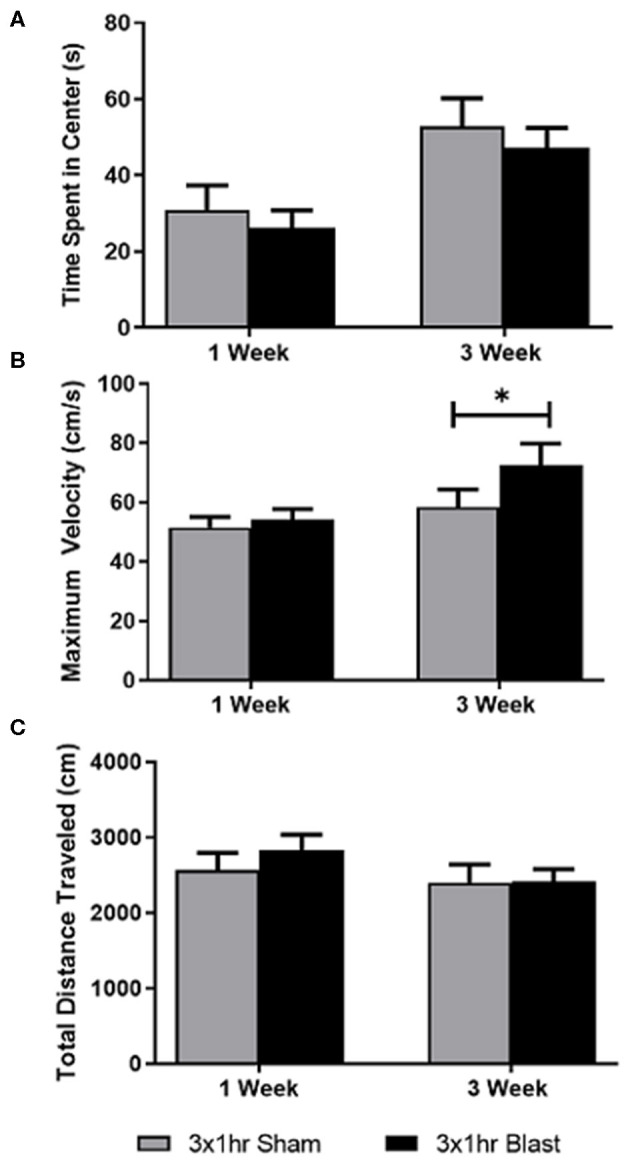
Testing anxiety-like behaviors following repeated bTBI. Using OFT, time spent in the center, maximum velocity, and total distance traveled were quantified at 1 and 3 weeks following injury. A two-way ANOVA with repeated measures was used to assess differences in the three parameters. **(A)** Time itself had a significant effect on the time spent in the center of the arena. No significance was found in the interaction between time and blast, or in treatment itself. **(B)** Analysis of maximum velocity indicated a significant difference between blast and sham groups at 3 weeks, but overall, blast and time alone did not reach significance. No significant interaction between time and blast was observed. **(C)** Significance in the interaction between time and blast was also not observed for total distance traveled. Significance in time and blast itself was also not found. *p < 0.05. Mean ± SEM.

The effect of multiple blast exposures was assessed using OFT at 1 and 3 weeks (Figure 3). Statistical analysis of the effects of repeated blast exposures on OFT performance depicted that time alone had a significant effect on the time spent exploring the center for blast and sham animals. Statistical analysis of the effects of repeated blast exposures on OFT performance depicted that time alone had a significant effect on the time spent exploring the center for blast and sham animals. No significance was observed in the interaction between time and blast (Figure 3A). A significant increase in the maximum velocity of blast animals was found at 3 weeks compared to shams. Even though this was observed, the overall treatment effect (blast) was not found to be significant. Time was also not found to be significant for maximum velocity (Figure 3B). No significance in interaction between time and blast was observed for total distance traveled, and time and blast alone was not found to be significant (Figure 3C). A summary of all behavioral analyses is found in Table 2.

Table 2Vestibulomotor deficits persist in repeated blast animals, while anxiety-like behavior is not detectable 1 and 3 weeks following injury.

**Table:** 

**Accelerating rotor rod**						
	**Latency to fall (s)**		**Distance traveled (cm)**		**Maximum RPM**	
	**One week**	**Three weeks**	**One week**	**Three weeks**	**One week**	**Three weeks**
Sham	79.93 ± 5.87	82.40 ± 7.12	151.7 ± 18.63	147.4 ± 23.09	21.80 ± 1.47	22.11 ± 1.57
Blast	67.26 ± 4.63^*^	67.40 ± 5.54^*^	111.2 ± 12.59^*^	111.9 ± 14.03^*^	18.64 ± 1.17^*^	18.73 ± 1.29^*^

**Table:** 

**Open field thigomotaxis**						
	**Time spent in center (s)**		**Distance traveled (cm)**		**Maximum velocity (cm/s)**	
Sham	26.64 ± 5.23	56.88 ± 6.93	2570 ± 225	2399 ± 243	51.58 ± 3.51	53.63 ± 3.90
Blast	26.16 ± 4.69	47.30 ± 5.13	2829 ± 210	2410 ± 167	54.11 ± 3.67	64.48 ± 6.19^*^

**Table:** 

**Accelerating rotor rod**					
**Latency to fall (s)**		**Distance traveled (cm)**		**Maximum RPM**	
**Source**	***p*****-value**	**Source**	***p*****-value**	**Source**	***p*****-value**
Time × Blast	0.8408	Time x Blast	0.8408	Time x Blast	0.9369
Time	0.8738	Time	0.7674	Time	0.884
Blast	0.0242	Blast	0.0223	Blast	0.0219

**Table:** 

**Open field thigomotaxis**					
**Time spent in center (s)**		**Distance traveled (cm)**		**Maximum velocity**	
Time × Blast	0.3651	Time x Blast	0.5595	Time x Blast	0.3464
Time	<0.0001	Time	0.1716	Time	0.163
Blast	0.4125	Blast	0.527	Blast	0.134

*Vestibulomotor impairment and anxiety-like behaviors were measured using RR and OFT at 1 and 3 weeks following repeated blast exposure. The results for the RR task were expressed as latency to fall, distance traveled, and maximum RPM. OFT data were expressed as time spent in the center, distance traveled, and maximum velocity. A two-way ANOVA with repeated measures for rotor rod indicated significance in treatment at 1 and 3 weeks, with no significance in the interaction between time and treatment or in time itself. OFT showed that time had a significant effect on time spent in the center at 1 and 3 weeks, with no significance in the interaction between time and treatment, or in treatment individually*.

* *p < 0.05, mean ± SEM*.

### Immunohistochemistry (IHC)

#### Elevated Levels of Microglia Found Within the Thalamus

**Figure 4:**
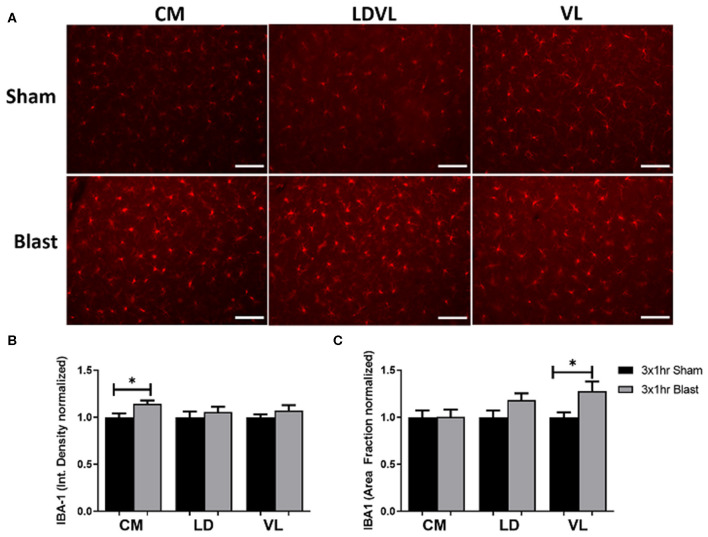
Increased IBA-1 expression and elevated levels of microglia following repeated blast exposure. **(A)** Elevated staining intensities of IBA-1 were observed in the blast animals as compared to the respective sham. Magnification is at 20x and scale bar = 100 μm. **(B)** The level of IBA-1 expression was quantified via the integrated density of fluorescence. A significant increase of IBA-1 was found in the CM region of the thalamus of blast animals compared to shams. Higher intensity of fluorescence may indicate activation of microglia following injury as upregulation of IBA-1 is found in activated cells. **(C)** The area fraction that the IBA-1 signal occupied signifies the percent of positive signal within a given region. A significant increase in the area fraction of IBA-1 was found in the VL region of the thalamus in the blast animals. A trending increase was noted in the LD region of the blast animals (0.0908). Increased area fraction may indicate proliferation of microglia to the injured regions. *n* = 10 per group. Mean ± SEM, *p < 0.05 and data was normalized to shams.

To identify areas of potential molecular mechanisms responsible for the observed vestibulomotor deficits, we examined the level of IBA-1 in three regions of the thalamus; CM, LD, and VL. IBA-1 is a common marker for microglia as it is involved in phagocytosis and actin reorganization in microglia. It is constitutively expressed in microglia and is elevated when microglia are activated in injuries such as blast (54–56). We therefore performed IHC and compared levels of IBA-1 in both blast and sham brains (Figure 4A). There was a significant increase (*p* < 0.05) in the bTBI compared to sham groups. Specifically, the integrated density of fluorescence was significantly increased in the CM region of the thalamus in blast animals in comparison to shams (Figure 4B). IBA-1 expression was also measured through the percentage of the positive signal within a given area (area fraction). There was a significant increase in area fraction of IBA-1 within the VL region of the thalamus for blast animals, with a trending increase in the LD (*p* = 0.0908) for blast animals compared to shams (Figure 4C). Quantification of averaged cell somas (mean area per cell) showed a trending increase in the CM in blast animals compared to the sham, with no significant differences found in the LD and the VL regions. There were no significant differences in the CM or VL regions of the thalamus between blast and shams in the number of cells per area of interest (count per area), with a trending increase in count per area in the LD region in blast animals compared to shams. The significant increase in IBA-1 expression between treatment groups suggests a compromised thalamus following repeated blast exposure.

Since the amygdala is associated with the sensorimotor complex, levels of IBA-1 in the BLA were measured. We found trending increases of IBA-1 in the integrated density of fluorescence in blast animals compared to sham (*p* = 0.0568). Increasing trends of IBA-1 were also observed in the area fraction (*p* = 0.0891) and mean area per cell (*p* = 0.0739) in blast animals. A summary of all IBA-1 analyses is found in Table 3.

**Table 3:** Increased levels of IBA-1 and GFAP expression can be found across thalamic regions.

	**Mean area per cell**		**Count per area**		**Integrated density**		**Area fraction**	
	**Mean ± SEM**	***p*-value**	**Mean ± SEM**	***p*-value**	**Mean ± SEM**	***p*-value**	**Mean ± SEM**	***p*-value**
**GFAP**								
CM Sham	7.941 ± 1.48	0.0239^*^	0.005 ± 0.001	0.1049	790147 ± 83864	0.3587	7.436 ± 1.36	0.0513
CM Blast	14.62 ± 2.265		0.011 ± 0.002		995887 ± 1998610		12.63 ± 1.91	
LD Sham	7.610 ± 0.57	0.079	0.059 ± 0.010	0.1708	560345 ± 44038	0.2614	11.83 ± 2.23	0.2734
LD Blast	9.649 ± 0.94		0.033 ± 0.010		648680 ± 60688		8.946 ± 1.25	
VL Sham	7.927 ± 1.09	0.2401	0.073 ± 0.020	0.6508	568916 ± 52308	0.627	11.58 ± 2.57	0.6727
VL Blast	9.96 ± 1.28		0.045 ± 0.014		611167 ± 67609		10.32 ± 1.45	
BLA Sham	58.73 ± 1.64	0.8305	0.0015 ± 0.001	0.5588	14557027 ± 986680	0.4879	8.879 ± 0.34	0.2491
BLA Blast	57.99 ± 3.40		0.0016 ± 0.001		13663037 ± 930213		8.608 ± 0.20	
**IBA-1**								
CM Sham	7.70 ± 0.33	0.0864	0.006 ± 0.002	0.3723	814061 ± 34270	0.0206^*^	4.41 ± 0.32	0.9794
CM Blast	8.74 ± 0.32		0.005 ± 0.002		930026 ± 30207		4.42 ± 0.35	
LD Sham	9.21 ± 1.13	0.8263	0.006 ± 0.001	0.0696	779684 ± 48406	0.5105	3.48 ± 0.25	0.0908
LD Blast	9.07 ± 0.48		0.005 ± 0.001		823351 ± 43806		4.116 ± 0.25	
VL Sham	8.81 ± 0.37	0.4537	0.005 ± 0.001	0.1444	951827 ± 30880	0.2977	3.96 ± 0.21	0.0283^*^
VL Blast	9.27 ± 0.47		0.006 ± 0.002		1020798 ± 54538		5.07 ± 0.39	
BLA Sham	129.4 ± 9.87	0.0739	0.004 ± 0.000	0.7859	937989216 ± 6193055	0.0568	13.67 ± 1.10	0.0891
BLA Blast	159.4 ± 12.84		0.004 ± 0.000		115647537 ± 85823062		18.27 ± 2.27	

Several parameters were measured for IHC in order to quantify both astrogliosis and microglia activation. Regions of the brain that are listed include the CM, LD, and VL regions of the thalamus, and the BLA region of the amygdala. Astrogliosis can be detected in all regions of the thalamus, with no significant differences or trends found in the BLA region of the amygdala. Microglia activation was also indicated in all three regions of the thalamus, with trending increases in IBA-1 expression found in the BLA region of the amygdala. All p-values represented were measured using the student t-test between the comparing of the sham and blast animal (n = 10 per group). Significance was observed at

* *p < 0.05 and trends were observed at p < 0.1 Data is also represented as Mean ± SEM*.

#### Astrocyte Reactivity Was Elevated Within the Thalamus

**Figure 5:**
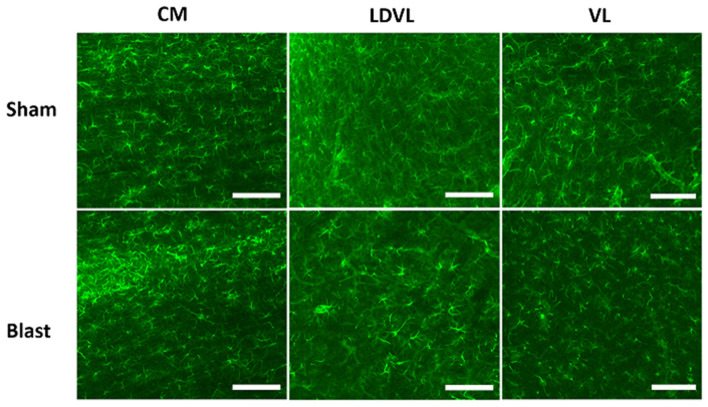
Representative images of GFAP obtained from the CM, LD, and VL regions of the thalamus. Magnification is at 20x and scale bar = 100 μm.

**Figure 6:**
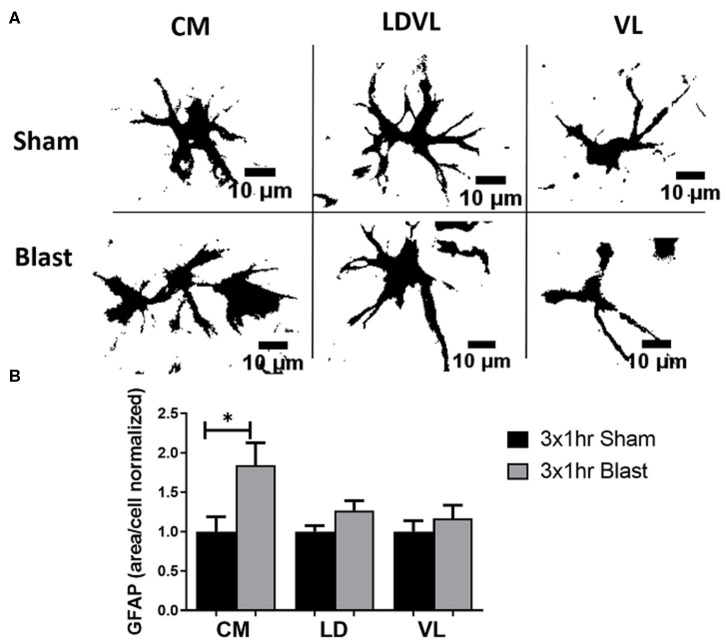
Significant astrogliosis after blast injury. **(A)** A view of individual astrocytes (GFAP) in both sham and repeated bTBI animals showed various sizes of cell bodies. Blast animals demonstrated larger areas of cells than shams. Scale bar = 10 μm. **(B)** A significant increase in GFAP mean area per cell was noted in the CM region of the thalamus of blast animals compared to shams. A trending increase in the mean area per cell was noted in the LD region (*p* = 0.079). This signifies that astrocytes are becoming reactive, changing size in response to injury. *p < 0.05, Mean ± SEM, data was normalized to shams.

To determine if the observed behavioral deficits are associated with astrocyte reactivity, we performed IHC to quantify the levels of GFAP in three regions of the thalamus; CM, LD, and VL (Figure 5). GFAP is an extensively studied biomarker of brain injury and has been commonly observed to be elevated in the brain following blast exposure. In rats exposed to repeated blast injury, there was prominent astrogliosis within the thalamus (Figure 6A). Astrocyte reactivity (area per cell) within the CM region was significantly increased (*p* < 0.05) due to repeated bTBI, with a trending increase in the LD region (*p* = 0.079). However, there were no significant differences observed in the VL region (Figure 6B). The increased soma of the astrocytes suggests changes in size (hypertrophy) taking place due to reactivity in response to the injured region. There were trending increases in the amount of GFAP signal (area fraction) and the count per area in the CM region of blast animals, however, there were no significant trends in integrated density in either regions of the thalamus. When examining the level of GFAP within the BLA, we found no significant differences in either of the parameters measured for GFAP, indicating limited astrocyte reactivity within the BLA at 1 month following injury. A summary of all GFAP analyses is found in Table 3.

## Discussion

In this study, we found vestibulomotor deficits in an early chronic phase of blast TBI. We showed that animals subjected to three repetitive blast events with an inter-blast interval of 1 h displayed decreased ability to complete the rotor rod tasks. These impairments were associated with glial pathology within the thalamus, with trends toward microglia activation taking place in the amygdala. Results from the study indicate that the blast exposures lead to long-term consequences that resemble those reported in Veterans with bTBI.

Few preclinical studies have reported on vestibular disorders following bTBI. Lien and Dickman investigated vestibular injury following low intensity blast exposure using the rotor rod behavioral task (10). They found a significant reduction in the ability of the animals to perform the balance task on the rotating rod for several weeks following blast exposure. In our current study, at 1 and 3 weeks following injury, blast animals showed significant decreases in RR tasks in comparison to sham animals, suggesting vestibular injury. Subsequently, there were pathological changes in the thalamus that may be linked to the vestibulomotor deficits seen in this behavior task. Elevated levels of IBA-1 in the thalamic nuclei suggests that there is an increase in the inflammatory response that aids in eliminating cell debris and the tissue repair process. Astrogliosis was also elevated in the thalamic nuclei. More specifically, the mean area per cell was increased in blast compared to sham animals, a sign of hypertrophy. As hypertrophy of astrocyte cell bodies and processes have been associated with reactive astrogliosis following blast exposure (57), this may indicate that these sequelae are taking place in the thalamus of blast-injured animals.

Limited studies on glial pathology within the thalamus following blast injury have been reported. A study by Studlack et al. focused on linking thalamic sensitization to headache and pain following blast injury (8). Their investigation included characterizing the astrocytic response within the VPM and the posterior thalamus (PO) which are associated with pain transmission. Brains examined 9 weeks following blast injury did not show elevated levels of astrocyte expression or reactive microglia in the PO or VPM. Further, no significant changes in gliosis within the PO was reported by a subsequent study (58). For both of these studies, the blast device/methods, behavioral assessments and timing of histological observation differed from our study, thus the results are difficult to compare.

Perez-Polo et al. characterized a rodent blast model using behavioral and neuropathological assays that included the thalamus and amygdala (59). They found that motor coordination (via beam-balance and foot-fault assays) was impaired following blast exposure. They reported elevated IBA-1 labeling in the thalamus as early as 6 h and lasting through 30 days following injury. Similarly, our group previously demonstrated increased astrocyte and microglia labeling in other brain regions such as the hippocampus up to 3 months following blast neurotrauma (43, 60). The results from this study suggest that glial activation in the rat thalamus may be similar to the gliosis occurring throughout the brain and likely influences the functional outcomes from this diffuse injury. Sajja et al. investigated amygdalar vulnerability following a single blast exposure (43). They found significant anxiety-like behaviors 1 week following a single bTBI that was associated with elevated levels of GFAP and IBA-1 within the amygdala. While the present study did not show significant anxiety-like behaviors 1 or 3 weeks following repeated blast injury, animals did present with trending increased levels of IBA-1 in the amygdala.

Although there is information identifying regional changes occurring within the brain, there is still a lack of understanding of how the affected areas work together to cause vestibular disorders. Injuries to the vestibular system can be related to pathology from the vestibular labyrinth of the inner ear to the transmission of the nerve impulses being carried by the vestibular nerve to many brain regions including the brain stem, thalamus, and cerebellum. Some fibers ascend into the vestibular area of the cerebral cortex after relaying information in the ventrolateral nuclei, laterodorsal nuclei, and central medial nuclei of the thalamus. The vestibular system itself contains many structures that are vulnerable to blast injuries (61). Damage to neuronal projections connecting the vestibular system with the thalamic and amygdala nuclei have been identified as contributing to vestibulomotor deficits seen as common sequelae of TBI (8, 62–64). While neuronal damage has been implicated, the mechanism of how glial cells contribute to the long-term recovery of the vestibular system is unknown. Moreover, astrocytes and microglia play important roles in the recovery of injured tissues. Reactive astrocytes work to return the brain environment back to its healthy state by balancing homeostatic deficits and mitigating oxidative stress. Activation of microglia and subsequent inflammatory response takes place as microglia accumulate and work to remove the extracellular debris or apoptotic cells following injury. At all stages of repair, glia help regulate the inflammatory response through the release of pro- and anti-inflammatory cytokines following injury.

Glial responses have been identified at the acute phase of injury following bTBI, however there is still limited knowledge on the sub-acute and early chronic outcomes, and how they contribute to vestibulomotor and behavioral deficits. Previous studies report on acute changes (2–72 h) of glial dysfunction (elevated levels of reactive astrocytes and activated microglia) in brain regions such as the hippocampus, amygdala, and the pre-frontal cortex (57, 60, 65). Sub-acute (3–14 days) results have also provided insight regarding the dynamic glial response occurring in the brain following blast injury (45, 66). Due to the gap in data from the early chronic stage of repair (longer than 14 days), this study was conducted to provide more evidence regarding how neurosensory changes, primarily glial dysfunction in the thalamus and amygdala, are contributing to vestibulomotor and behavioral deficits.

Clinical studies have indicated that thalamic damage transpires following both blast and impact-related injuries and have an impact on motor and cognition impairments (67). Neurosensory outcomes such as vestibulomotor impairment have become increasingly common in military personnel, Veterans and civilians diagnosed with TBI (1, 61). However, those exposed to blast events appear to have a unique set of outcomes compared to those involved in impact-related injuries. Hoffer et al. conducted a clinical study to investigate this premise (68). They examined the vestibular-ocular and vestibular-spinal reflexes in two separate cohorts of mild TBI (mTBI) patients; blunt and blast head trauma. They found that a higher percentage of blast-exposed patients exhibited a trend toward low-frequency phase lag on evaluation. A subsequent study completed by the same group was completed that studied active military personnel exposed to blast (69). They performed vestibular function and auditory tests, subsequently compared results to those with impact-related head injuries. They found that vestibular function significantly worsened in blast-exposed patients as a function of time between injury and presentation. They also noted that the blast group presented with a unique set of vestibular disorders and associated symptoms as compared to the impact-related group. These studies identify a distinct difference between impact and blast injured mTBI patients and provide evidence that treatment strategies should be individualized on the basis of each mechanism of injury. This suggests that the mechanism of vestibular injury differs between blast and impact-related TBI. Understanding the differences between these two distinct types of injury would lead to a more focused approach by clinicians to develop better treatment strategies for those exposed to blast injury.

The debate on the biomechanical transmission of blast wave energy to the brain is also ongoing, but many accept that the injury mechanism differs from impact-related head injuries. Explanations point to a dynamic skull deformation theory that produces high-speed compression leading to shear stress between fluid and tissue interfaces (70). Brain tissue at interfaces with fluid, such as that bordering the cerebrospinal fluid-filled ventricles or blood-filled sinuses, are thought to be particularly susceptible to primary blast injury due to the reflection of blast waves at borders of materials with differing densities. This stress likely contributes to the cellular response triggered by blast exposure. While areas of the thalamus may be protected from the fluid-tissue interface stress as its location is in the center of the brain, transmission of compressive forces may also explain the inner brain region damage seen in some models. Animal models of both single and repeated blast exposure also indicated neuronal injury and glial dysfunction due to the biomechanical stress transmission.

As we advance the generation of clinically relevant data to decipher blast injury mechanisms, we will be able to assist in further understanding the differing outcomes and neuropathy observed between blast and impact injury modes, bTBI is characterized as a diffuse injury that presents with persistence gliosis (71). A limitation of the current study is the use of IBA-1 to detect microglia in the brain. IBA-1 has an affinity to both microglia and monocyte-derived macrophage surface markers (72–74), thus the use of IBA-1 cannot fully distinguish between the local and systemic inflammatory response. The timing of when the systemic macrophage resolve from the brain injury is debated. Studies have emerged reporting that CCR2-dependent macrophages are recruited from the periphery then dissipate in the early chronic stages of injury (75, 76). These reports support the hypothesis that the key players of the inflammatory response 4 weeks after injury is the resident microglia and astrocytes, making glial contributions the focus of our studies. Future work should test this hypothesis by using microglia-specific markers for a confirmation of glial characterization following bTBI. Transmembrane 119 (TMEM 119) is an example of a recently identified microglia marker that has been used by multiple sclerosis researchers and could be applied to TBI studies (77). Collectively, the current study has proven that glial activation within the thalamus is contributing to the ongoing vestibulomotor deficits following blast induced injury, especially at the chronic stages. We hope that the advancement of these studies will lead to further strategies that will aid in long-term healthcare for bTBI patients, ultimately improving their quality of life.

## Data Availability Statement

All datasets presented in this study are included in the article/supplementary material.

## Ethics Statement

The animal study was reviewed and approved by Virginia Tech Institutional Animal Care and Use Committee.

## Author Contributions

MD was responsible for analysis of results, interpretation of results, and preparation of manuscript. ZB was responsible for data collection, analysis of data, and interpretation of results. SM and MU was responsible for data collection and analysis of data. PV was responsible for study design, securing funding, interpretation of results, and preparation of manuscript. All authors contributed to the article and approved the submitted version.

## Conflict of Interest

The authors declare that the research was conducted in the absence of any commercial or financial relationships that could be construed as a potential conflict of interest.
